# Birth of two volcanic islands in the southern Red Sea

**DOI:** 10.1038/ncomms8104

**Published:** 2015-05-26

**Authors:** Wenbin Xu, Joël Ruch, Sigurjón Jónsson

**Affiliations:** 1 King Abdullah University of Science and Technology (KAUST), Thuwal 23955, Saudi Arabia

## Abstract

Submarine eruptions that lead to the formation of new volcanic islands are rare and far from being fully understood; only a few such eruptions have been witnessed since Surtsey Island emerged to the south of Iceland in the 1960s. Here we report on two new volcanic islands that were formed in the Zubair archipelago of the southern Red Sea in 2011–2013. Using high-resolution optical satellite images, we find that the new islands grew rapidly during their initial eruptive phases and that coastal erosion significantly modified their shapes within months. Satellite radar data indicate that two north–south-oriented dykes, much longer than the small islands might suggest, fed the eruptions. These events occurred contemporaneously with several local earthquake swarms of the type that typically accompany magma intrusions. Earthquake activity has been affecting the southern Red Sea for decades, suggesting the presence of a magmatically active zone that has previously escaped notice.

## Abstract (web summary)

How new volcanic islands form is not well understood, particularly those along mid-ocean ridge systems. Here, the authors present observations of two new islands that were formed in the southern Red Sea, using high-resolution satellite imagery to quantify their growth and subsequent erosion.

## Graphical abstract

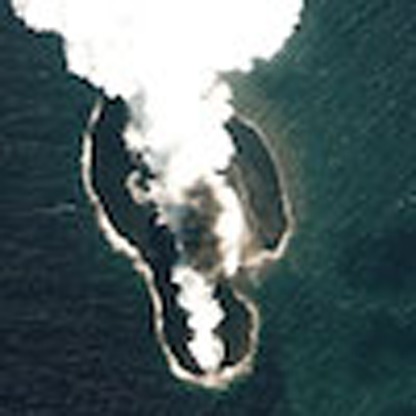

Together with the Gulf of Aden and the African rifts, the Red Sea accommodates the divergence between the Nubian, Somalian and Arabian plates from the Afar triple junction^1^. The southern Red Sea rift is divided into two branches south of 17°N, separated by the Danakil block, with the Danakil depression to the west and the southernmost Red Sea ridge to the east^2^,^3^ (Fig. 1a). Regionally, the triple-junction has been subject to intense magmatic activity since at least the late 1970s, underscored by three major rifting episodes (Asal-Ghoubbet, 1978; Dabbahu 2005–2010; Gulf of Aden 2010 and 2011) and at least four single-diking events^4^,^5^,^6^,^7^. Most of this activity has occurred on shore, where geodetic data have provided unambiguous evidence of magmatic intrusions, with the exception of the December 2010 Gulf of Aden seismic sequence. In that offshore event, the seismic activity was interpreted as symptomatic of a magmatic intrusion, characterized by earthquake swarms that lack mainshock–aftershock decays and exhibit a roughly constant seismicity rate^6^,^8^,^9^. Such seismic swarms typically span from a few hours to a few weeks with peak earthquake magnitudes usually ranging from M3 up to M5. In the absence of surface evidence (that is, eruptions or deformation), most intrusions along mid-ocean ridge systems likely remain overlooked.

A significant increase in seismicity was observed in the southern Red Sea in 2007, before an eruption that took place on Jebel at Tair Island^10^. The Zubair archipelago, which is located roughly 50 km to the southeast on the southern Red Sea ridge, has seen similar seismic swarm sequences during the past few decades (ISC catalogue Fig. 1a,b). Although earthquake locations are not accurate in the southern Red Sea, the timing and the seismic moment release provide valuable information about the activity. At least six seismic swarms have occurred in the past 20 years, probably resulting from separate magma intrusions. Three of them, in 2007, 2011 and 2013, were followed by eruptions within a year (Fig. 1b).

The Zubair archipelago is composed of ten volcanic islands and several rocks located within the central part of the southern Red Sea ridge, between Yemen and Eritrea (Fig. 1a,c). These islands and rocks are the visible manifestations of a 30-km-long and 10-km-wide shallow (<100 m) platform oriented parallel to the Red Sea ridge. At this latitude, the spreading rate that separates the Arabian Plate from the Danakil block is ∼6 mm per year in the northeast direction^3^. These islands are the only surface evidence of active volcanism along the entire Red Sea, together with the Jebel at Tair Island and the Hanish-Zukur islands (120 km to the southeast; Fig. 1a)^11^. The Zubair archipelago is dissected by faults, fracture systems and several eruptive fissures (for example, on the Zubair, Center Peak and Saba islands). The mean orientations of these features, determined from high-resolution optical imagery, show an overall dominant north–south orientation, somewhat oblique to the Red Sea ridge (Fig. 1c). A few eruptions are known to have occurred on the Jebel at Tair Island and within the Zubair archipelago in the eighteenth and nineteenth centuries^11^. However, more than a century of apparent quiescence followed that activity until eruptions occurred on Jebel at Tair in 2007–2008 and few years later in the Zubair archipelago^10^,^12^.

Witnessing the birth and evolution of new volcanic islands is exceptionally rare, particularly along divergent plate boundaries. Here we use high-resolution satellite optical and radar imagery to study these new remote islands as well as to map the co-eruptive deformation on neighbouring islands. Together with observed seismicity in the region, we show that the southern Red Sea ridge has been magmatically active for at least two decades, supporting the presence of an active spreading centre.

## Results

### The 2011–2012 Sholan eruption

**Figure 1:**
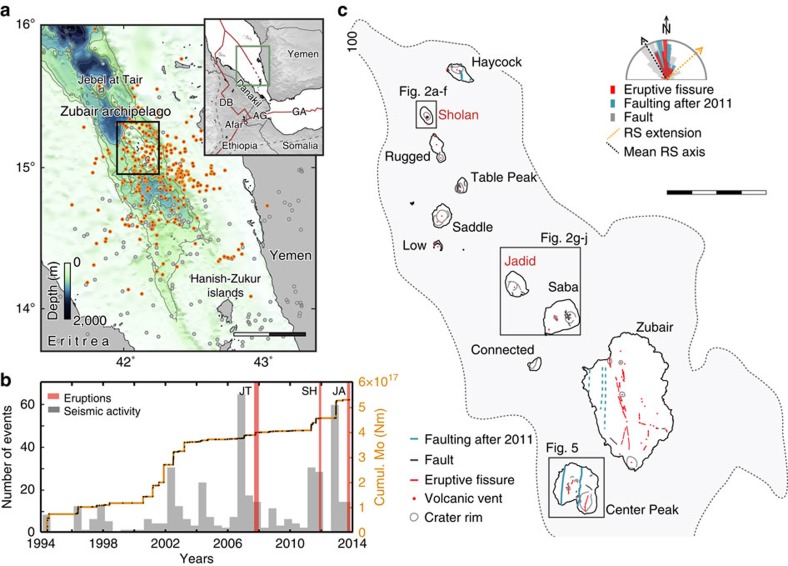
Zubair archipelago and its seismic and volcanic activity in 1994–2014. (**a**) Earthquake locations (ISC catalogue) in the southern Red Sea with orange dots highlighting events related to seismic swarms. Red lines in the inset delineate the plate boundaries^36^ separating the Nubian, Somalian and Arabian plates. DB, Dabbahu; AG, Asal-Ghoubbet; GA, Gulf of Aden. Scale bar, 50 km. (**b**) Cumulative seismic moment release (orange line), number of events per 3 months (grey bars) and eruptions (red bars) at Jebel at Tair (JT), Sholan (SH) and Jadid (JA) islands. (**c**) Map of the Zubair archipelago (the grey area marks platform depths shallower than 100 m) including the new Sholan and Jadid islands and general structural feature locations and orientations (stereoplot; RS for Red Sea). Scale bar, 4 km.

**Figure 2:**
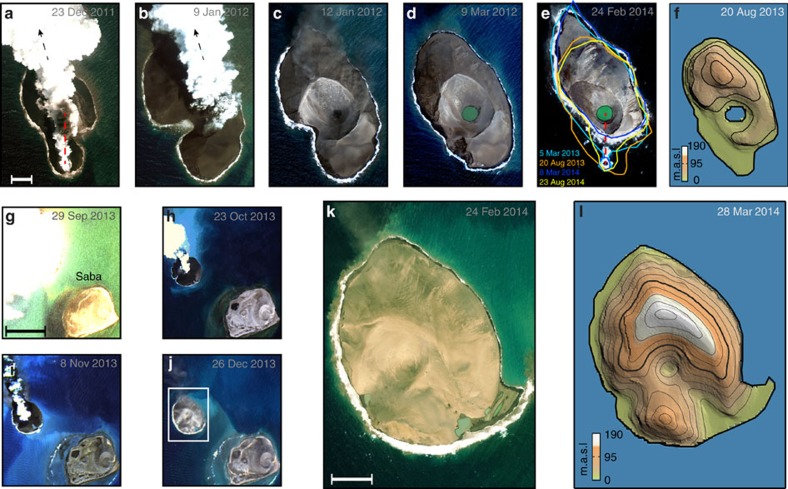
Formation and erosion of the new volcanic islands. High-resolution optical satellite images show (**a**–**b**) the 2011–2012 Sholan eruption and (**c**-**e**) its post-eruptive changes (scale bar in (**a**), 100 m) and (**g**-**i**) the 2013 Jadid eruption (scale bar in (**g**), 1 km) and (**j**) its post-eruptive change. (**k**) Zoom-in of Jadid island from a WorldView-2 image. Scale bar, 200 m. (**f**) and (**l**), TanDEM-X shaded relief topography of Sholan and Jadid islands with 20-m elevation contours. The red dashed line in **a** and **e** indicates the 2011–2012 eruptive fissure orientation; the black dashed arrows in **a** and **b** indicate the direction of volcanic ash deposits during the construction of the island, and coastline changes are colour-coded in **e**.

The first of the two Zubair eruptions took place on the shallow platform between Haycock and Rugged islands in the northern part of the archipelago in December 2011 (Fig. 1c and Supplementary Fig. 1, refs 13, 14). Three distinct earthquake swarms in April, June and August 2011 occurred in the area during the eight months before the eruption (3<M<4.5; Fig. 1b), followed by two earthquakes (magnitude 3.7 and 3.9) detected by the Yemeni seismological network on the mainland ( http://www.nsoc.org.ye) on 13 December 2011. Yemeni fishermen reported an eruption in the Zubair archipelago on 18 December and on the following day a SO_2_ anomaly can be seen in an Ozone Monitoring Instrument satellite image ( http://www.volcarno.com/). A high-resolution WorldView-2 satellite optical image from 23 December reveals that a new island (hereafter called Sholan Island) had formed following a short Surtseyan eruption (Fig. 2a). Initially, the eruption appears to have been fed by a short north–south-oriented fissure (Fig. 2a), somewhat oblique to the southern Red Sea ridge, but consistent with the mean eruptive fissure trends of the entire Zubair archipelago (N354±5°; Fig. 1c). The activity later condensed to a single vent (Fig. 2b) and the island grew until the eruption ended on 12 January 2012 (Fig. 2c). The full duration of the eruption was 25 days. The eruptive activity was influenced by the inflow of seawater and did not evolve from an explosive to an effusive eruption. In addition, steady southeasterly winds influenced the shape of the island during its construction, such that the main deposits fell obliquely to the orientation of two active vents (Fig. 2a). By the end of the eruption, Sholan Island had grown to a maximum width and length of 0.52 and 0.77 km, respectively, with a subaerial area of 0.25 km^2^. Yemeni scientists visited the new island 5 days after the end of the eruption and found that it consisted of hydromagmatic deposits. They witnessed several small-scale landslides on the steep and unstable flanks of the island (Jamal Sholan, personal communication 2013).

Rapid coastal erosion is observed on the island's southern shore in post-eruption images, with the island losing ∼0.01 km^2^ of its subaerial area during the first 2 months following the end of the eruption (Fig. 2c,d). In addition to the coastal erosion, a crater lake formed, suggesting that highly permeable deposits were infiltrated by seawater. Wind erosion also contributed to the observed morphological changes; loose unconsolidated eruptive products are seen blowing away from Sholan and surrounding islands in several post-eruption optical images (Fig. 2c-e). As a result of seasonally varying wind and ocean current directions, the island grows to the north in the winter and spring, while deposits move back from the northern shore to the southern part of the island in the summer (Fig. 2e and Supplementary Fig.2). By 24 February 2014, the subaerial area of Sholan Island had decreased by ∼30% to 0.18 km^2^.

### The 2013 Jadid eruption

A second submarine eruption began on 28 September 2013 between the Saddle and Saba islands ( http://www.volcarno.com/), ∼19 months after and 8 km southeast of the Sholan eruption (Fig. 1c). An earthquake swarm was recorded in December 2012, followed in May and September 2013 by two additional swarms (Fig. 1b). Six earthquakes with magnitudes <4 were detected to the east of Zubair islands 2 days before the eruption was first noticed. An optical Landsat-7 image acquired on 29 September 2013 shows discoloured seawater and a thick plume rising from the sea surface (Fig. 2g). A new island is seen in an image from 23 October when a prominent, sub-circular, 0.8-km-diameter cone had formed (hereafter called Jadid Island; Fig. 2h). Additional imagery shows that the eruption continued until 20 November 2013 (ref. 12). Similar to the earlier activity, this eruption did not evolve into an effusive eruption, even though its duration was significantly longer (54 days) than the previous eruption. At the end of the eruption, the Jadid Island had grown to a near circular shape with a diameter of ∼0.9 km and a subaerial size of ∼0.68 km^2^ (Fig. 2j), more than twice that of Sholan Island. Erosion at Jadid Island has been less pronounced than at Sholan, but coastal erosion can still be observed at the southern shore in post-eruption images (Fig. 2k and Supplementary Fig. 3). By 24 February 2014, the subaerial size of the island had slightly decreased to 0.67 km^2^.

### InSAR observations and modelling

Interferometric synthetic aperture radar (InSAR) data from the TerraSAR-X and TanDEM-X radar satellites enable us to generate high-resolution digital elevation models (DEMs) of the newly formed islands^15^. We combined data from both the descending and the ascending orbits to generate the DEMs, which show that the subaerial height and volume of Sholan Island was about 94 m and 0.0057, km^3^, respectively, in August 2012 (Fig. 2f). During the following 2 years, Sholan Island lost a significant part of its area and volume to erosion (Fig. 2). In comparison, the height and volume of Jadid Island were found to be ∼186 m and 0.047 km^3^, respectively, in March 2014 (Fig. 2l).

**Figure 3:**
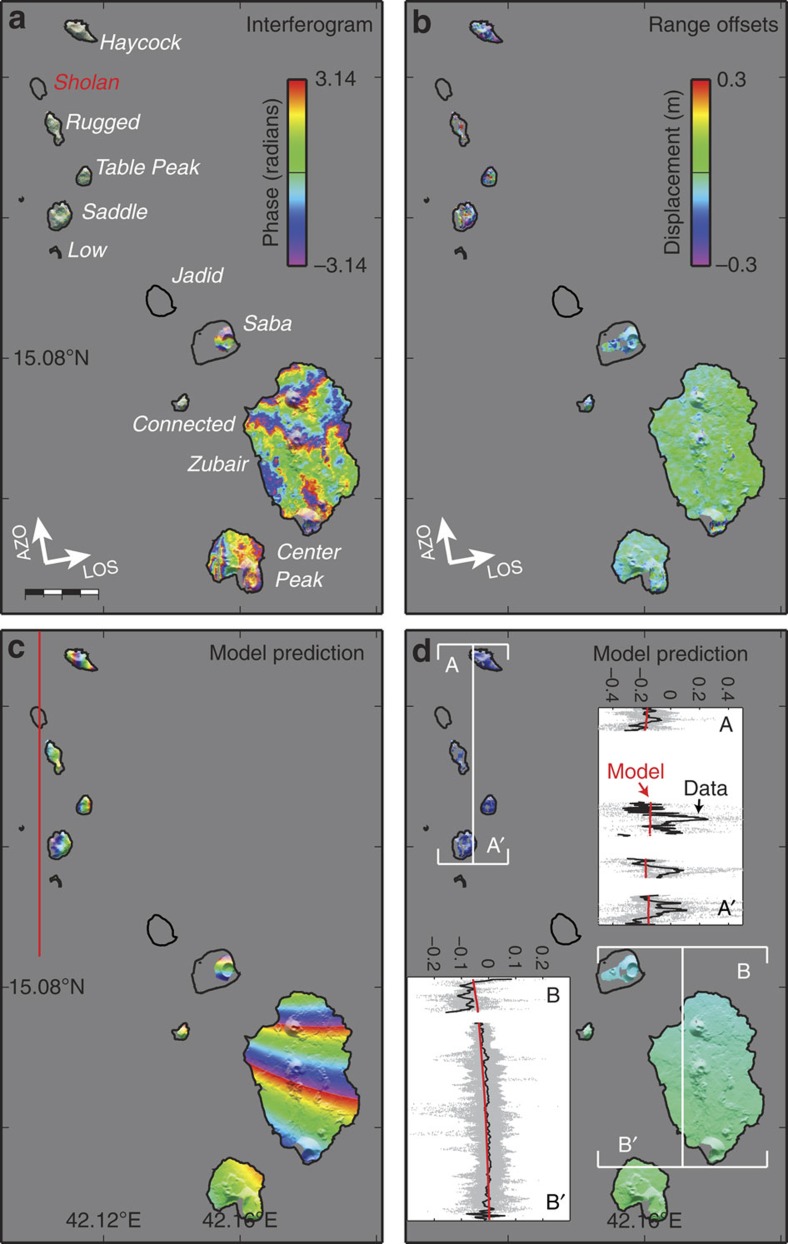
Co-eruptive deformation of the 2011–2012 Sholan eruption. (**a**) InSAR data (from an ascending orbit), (**b**) range offsets (**c**) and (**d**) model predictions for **a** and **b**. The red line in **c** marks the surface projection of the modelled dyke under the Sholan Island. Insets in **d** show observed (black) and modelled (red) displacements along profiles A-A' and B-B'. Negative range-offset values in **b** and **d** indicate the ground motion away from the satellite. Scale bar in (**a**), 2 km.

InSAR data were also used to measure centimetre-scale ground deformation associated with the eruptions^16^,^17^. The only available interferogram (13 October 2011–15 December 2012) bracketing the 2011–2012 Sholan eruption shows at least one fringe of deformation (1.5 cm) on Saba Island and about two fringes on Zubair Island (Fig. 3). In addition, localized signals are observed on Connected Island (∼4 cm in the line-of-sight direction), located ∼11 km southeast of Sholan, and at least 3 cm of normal-faulting movement along a north–south-oriented fracture on the Center Peak Island, which is ∼15 km from the eruptive activity. While the interferogram provides limited information about the northern Zubair islands due to decorrelation (probably, at least in part, due to blanketing tephra deposits), several irregular InSAR fringes are observed on Haycock Island (Fig. 3), just 2 km northeast of the eruption site. A new fracture system oriented north–northwest to south–southeast is also seen on this island in an optical image acquired on 23 December 2011 that is not present in images from before the eruption. These open fractures appear to be at least 0.5 m wide, estimated from the optical imagery pixel sizes. When taken together, the satellite data indicate widespread deformation, suggesting that the entire Zubair archipelago was affected during the eruption.

**Figure 4:**
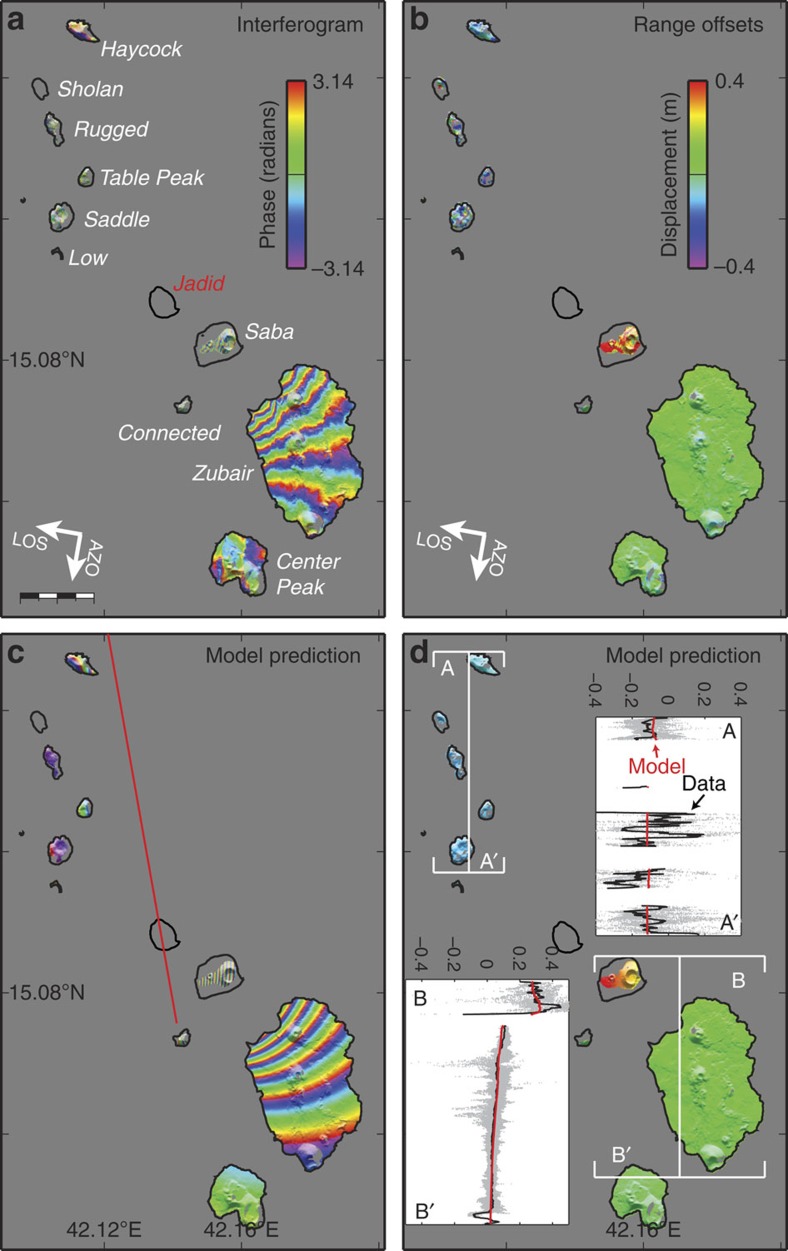
Co-eruptive deformation of the 2013 Jadid eruption. (**a**) InSAR data (from a descending orbit), (**b**) range offsets (**c**) and (**d**) model predictions for **a** and **b**. The red line in **c** marks the modelled dyke under Jadid Island. Insets in **d** show observed (black) and modelled (red) displacements along profiles A-A' and B-B'. Positive range-offset values in **b** and **d** indicate the ground motion towards the satellite. Scale bar in (**a**), 2 km.

We also generated two interferograms (from ascending and descending orbits) that span the 2013 eruption at Jadid Island. Both interferograms show several continuous InSAR deformation fringes across Saba and Zubair islands (Fig. 4a and Supplementary Fig. 4a), indicating that the co-eruptive ground deformation extended far beyond Jadid Island. In addition, range offsets^18^ between the SAR images (see Methods section for details) show that the northern islands moved towards the northwest while Saba Island was displaced to the southeast (Fig. 4b). This deformation pattern suggests that the dyke that fed the eruption was emplaced between the islands.

**Figure 5:**
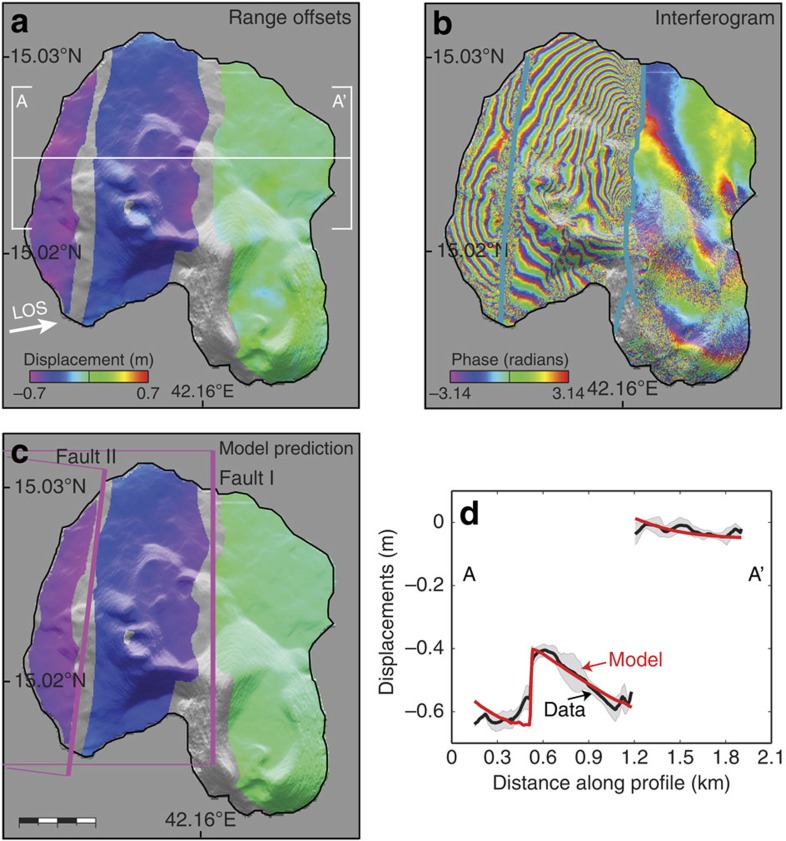
Normal faulting on Center Peak Island. (**a**) TanDEM-X range offsets (from an ascending orbit, motion away from the satellite shown negative), (**b**) InSAR data and (**c**) modelled displacements. Blue lines in **b** show fresh surface fractures and purple rectangles in **c** surface projections of the two model faults, with thicker lines marking fault's upper edges. (**d**) Observed and modelled displacements along profile A-A' in (**a**). Scale bar in (**c**), 0.4 km.

One of the two interferograms (ascending orbit, 15 December 2012–24 December 2013) spanning the Jadid eruption shows a significant amount of ground deformation near two north–south-oriented displacement discontinuities or faults on the Center Peak Island, located ∼8 km south of the eruption site (Fig. 5a). The western discontinuity is the same as the one seen moving in the InSAR data of the 2011–12 Sholan eruption. We measured the fault-offset displacements as exceeding 60 and 20 cm for the eastern and western discontinuities, respectively (Fig. 5c). The other interferogram (descending orbit, 20 August 2013–28 March 2014) bracketing the Jadid eruption does not show clear faulting movement, indicating that the bulk of this deformation occurred between 15 December 2012 and 20 August 2013. This faulting may therefore have occurred during the strong seismic swarm in December 2012 (Fig. 1b); that is, between the two eruptions.

To analyse the cause of the deformation, we constrained parameters of models using both the InSAR observations of the southern Zubair islands (that is, the Saba, Zubair, Connected and the Center Peak Islands) as well as range offsets of the whole archipelago. A north–south-oriented, ∼10 km-long feeder dyke (∼1.5 m thick) under Sholan Island appears to explain well the observed deformation in the earlier eruption (Fig. 3, Table 1). We found that the fringes observed in the 2013 Jadid eruption on both Saba and Zubair islands and the range offsets are best modelled by a dyke intrusion under Jadid Island that is ∼12-km long, trending north–northwest (350°) and with a thickness of ∼1.0 m (Fig. 4, Table 1). This dyke model, however, does not explain the ascending interferogram well (Supplementary Fig. 4), which might be due to an additional deformation in that interferogram caused by the seismic swarm in December 2012 or related to strong atmospheric signals. In addition, we model the observed faulting on the Center Peak Island with two north–south-striking, west-dipping normal faults, oblique to the southern Red Sea ridge axis (Fig. 5).

## Discussion

Our results confirm that the southern Red Sea is an active plate boundary separating the Danakil bock from the Arabian plate, which probably continues southward, in the direction of the Hanish-Zukur group of islands (Fig. 1a). This has also been inferred by GPS data, which shows a rate of opening of ∼6 mm per year at this latitude^3^. Together with the Danakil depression, located ∼200 km to the west, the southern Red Sea ridge therefore contributes to the total plate divergence of 16 mm per year between the Arabian and the Nubian plates.

Our satellite data and modelling suggest that the magma intrusions feeding the Zubair eruptions both took place along the same north–south-oriented fracture system, with the dykes separated laterally by ∼4 km. This possibility is supported by co-eruptive fractures and faulting on Haycock, Zubair and the Center Peak islands with similar orientations (see Fig. 1c), mapped using both high-resolution optical and InSAR images. In terms of size, morphology and seismic activity, the Zubair archipelago is comparable with active inland spreading centres, such as those found in Afar or in Iceland^19^. The archipelago's offshore location and poor bathymetric resolution do not allow for a detailed morphological analysis; however, systematic surface faults on most of the islands support the presence of a possible fissure swarm, inherently associated with the spreading centres.

In terms of total seismic moment release, the December 2012 earthquake swarm corresponds to a single M5.2 event, whereas a significantly larger moment release would be needed to explain the geodetic observations of the fault displacements on the Center Peak Island (at least M5.2 and M5.6). It is therefore likely that another dyke was emplaced below the island in December 2012, causing the large, partly aseismic faulting displacements (Fig. 5). In this scenario, the three latest intrusions at Zubair suggest the possible alternating intrusion locations, where a new intrusion occurs where the tensile stress generated by the previous intrusion is expected to be the highest (that is, at the dyke tips). Similar intrusion patterns were observed during the latest rifting episodes at Krafla in Iceland and Dabbahu in Ethiopia^20^,^21^ with the entire areas suffering from many seismic swarms, indicating that multiple magma intrusions occurred. The temporal and spatial pattern of intrusions within the Zubair archipelago is overall comparable to other rifting episodes, which are characterized by a series of dyke intrusions occurring over a period of several years^22^. We thus suggest that the recent volcanic and seismic activity may be due to a rifting episode in the Zubair archipelago.

From a structural point of view, the main fracture zones, the eruptive fissures and the new dykes affecting the entire archipelago are all oriented oblique by 10–20 degrees clockwise to the southern Red Sea ridge (see Fig. 1c). Similar recent (<1 Ma) fault orientations have been detected over the oceanic crust located beneath the Farasan islands, 200 km to the north^23^, and regional off-rift volcanism (for example, Harrat Rahat) shares a similar intrusion orientation^24^,^25^. There is no clear explanation yet for the apparent obliquity between the Zubair structures and the southern Red Sea ridge. A steep-sided trough marks the ridge, reflecting a zone of profound crustal stretching, and the Zubair islands lie near the eastern border of the trough (Fig. 1c). Rift obliquities are in some cases observed between the main rift-border faults and the active intrusion zones, as along the Main Ethiopian Rift^26^. In such cases, the obliquity may be explained by a change in rift kinematics that further focuses magma upwelling^27^ or it could alternatively relate to rift segmentation controlled by rift obliquity alone^26^. Thick evaporitic sediments (up to 2-3 km) covering the southern Red Sea ridge hide most of the structures in the surroundings of the Zubair Islands, making further interpretations difficult. We note, however, that the Zubair islands are located over a large regional low-velocity anomaly, which has a north–south orientation and extends to the north under the entire Arabian shield^28^, interpreted as being an offset mantle flow from the opening of the Red Sea. In this specific case, the magmatic activity and the intrusion geometry in the Zubair archipelago might be the surface expression of deep mantle flow interacting with the southern Red Sea ridge.

While the InSAR and seismicity data do not constrain the intrusion depth well and therefore do not provide accurate information about the magma plumbing related to this spreading centre, they do show that the eruptions were fed by dyke intrusions that were much larger than the small size of the two new islands might suggest. The challenge of modelling the ground deformation primarily comes from the limited emerged land surface, introducing inevitably additional uncertainties. For the 2011–2012 Sholan eruption, the northern group of islands, however, provides some constraints for the strike and location of the feeder dyke. Furthermore, the location of Sholan Island, the fringe patterns observed on the southern islands, and the orientation of the erupting fissure provide key information to constrain the location and length of the feeder dyke. Similarly for the 2013 eruption, the location of Jadid Island, the fringe patterns observed on the southern islands and the range offsets of the whole archipelago allow us to bind the location, length and strike of the feeder dyke in the modelling.

When the Surtsey eruption in Iceland ended in June 1967 after 4 years of activity, the island had reached an area of 2.7 km^2^ and a height of 175 m above sea level. Significant coastal erosion has been observed since then. In 1975, its size had decreased to 2 km^2^ and by 2012, the area of Surtsey had been reduced by half, to an area of 1.3 km^2^ (ref. 29). Loose tephra on Surtsey Island altered into palagonite tuff after the eruption, which has been found to be much more resistant to erosion than its fractured lava^30^. In the southern Red Sea, the wind and ocean currents are less severe than those south of Iceland; even so, they have heavily eroded the southern part of the Sholan Island, reducing its area by 30% in only 2 years. Jadid Island, however, seems to be more resistant to erosion than is Sholan Island and has retained almost all its surface area. Similar to Surtsey Island, many of the older and smaller Zubair islands consist of tuff that has not easily eroded away. This is likely also going to be the fate of the new Jadid and Sholan islands, that is, they are going to remain above the surface despite the fast erosion observed immediately after the eruptions.

By combining high-resolution optical imagery, InSAR observations and seismicity, we characterize with unprecedented details the birth and development of two volcanic islands along a mid-ocean ridge system. Our results show that the southern Red Sea has been magmatically active for several decades and that intrusions affect the entire Zubair archipelago, far beyond localized eruption sites. These results, together with the overall morphology of the platform, suggest that Zubair is the surface expression of an active spreading segment that was previously under appreciated.

## Methods

### InSAR data processing for the both eruptions

We used two ascending TerraSAR-X images from 13 October 2011 and 15 December 2012 to generate the co-eruption deformation map of the 2011–2012 Sholan eruption (Supplementary Table 1). The perpendicular baseline of this interferogram is ∼20 m. The two co-eruptive interferograms spanning the Jadid eruption were processed using descending TerraSAR-X images (20 August 2013 and 28 March 2014) and ascending TerraSAR-X (15 December 2012) and TanDEM-X (24 December 2013) images. The perpendicular baseline is ∼25 m in both the cases.

We processed the data with the GAMMA software and used our TanDEM-X DEM to simulate and eliminate the topographic signals. The interferogram noise was first reduced by ‘multilooking' and then by filtering^31^. The interferograms were then unwrapped using the minimum cost flow method^32^ and finally geocoded into the WGS84 coordinate system. We also calculated spatial-variable offsets between the SAR images^18^, both for the line-of-sight slant-range and azimuth directions. The north–south-oriented dykes produced limited deformation in the azimuth direction, so we used only the range offsets (along with the InSAR data) in the modelling. The accuracy of slant-range offsets is typically of the order of 1/10th of a pixel^18^, that is, ∼14 cm in slant-range for the TerraSAR-X data. Unlike the InSAR phase, the offsets provide unambiguous estimates of the relative displacement between the islands. We therefore used the range offsets to correct the phase unwrapping ambiguities between the islands.

### Deformation modelling

To model the observed deformation, we used rectangular dislocations in homogeneous, isotropic and elastic half-spaces^33^ to represent the feeder dyke intrusions and normal faults. The InSAR data were subsampled with a quadtree method^34^ and a simulated annealing algorithm^35^ was used to constrain the optimal dyke geometry and opening.

We first modelled the 2013 Jadid feeder dyke using data from both the ascending and descending orbits, primarily the InSAR data covering the southern islands and range offsets covering the whole archipelago (Fig. 4 and Supplementary Fig. 4). In the modelling, we used a 170°-oriented dyke and constrained it to be vertical and pass through Jadid Island. A north–south-oriented dyke predicts similar deformation but causes larger r.m.s. misfit than the 170°-oriented dyke model. We therefore selected the 170°-oriented dyke model to represent the Jadid feeder dyke. Following a similar procedure, we modelled the dyke that fed the 2011–2012 Sholan eruption using InSAR data covering the southern islands (Saba island, Zubair island and Center Peak island) and range offsets covering the whole archipelago. We fixed the dyke orientation to be north–south, according to the fissure orientation seen in optical images (Fig. 2a) and further constrained it to be vertical and to pass through the Sholan Island.

## Additional information

**How to cite this article:** Xu, W. *et al.* Birth of two volcanic islands in the southern Red Sea. *Nat. Commun.* 6:7104 doi: 10.1038/ncomms8104 (2015).

## Supplementary Material

Supplementary Information Supplementary Figures 1-4 and Supplementary Table 1

## Figures and Tables

**Table 1:** Estimated model parameters for the dykes feeding the 2011–12 Sholan and 2013 Jadid eruptions and for the faulting observed on the Center Peak Island, likely associated with the December 2012 earthquake swarm.

**Name**	**Lat (deg)**	**Long (deg)**	**Length (km)**	**Width (km)**	**Depth (km)**	**Strike (deg)**	**Dip (deg)**	**Dip-Slip (m)**	**Thickness(m)**	**M**_**w**_
Dyke 2011	15.145	42.101	10	2.1	0	360	90	0	1.5	—
Dyke 2013	15.126	42.132	12	2.1	0	350	90	0	1.0	—
Fault I	15.024	42.161	1.7	6.6	0	0	76	0.9	0	5.6
Fault II	15.023	42.154	1.7	2.3	0	7	63	0.6	0	5.2

Abbrevations: deg, degree; Lat, latitude; Long, longitude; M_w_, moment magnitude.
