# Supplementary material for: Birth of two volcanic islands in the southern Red Sea
Source: Nat Commun. 2015 May 26;6:7104. doi: 10.1038/ncomms8104 (PMC4455101; doi:10.1038/ncomms8104)
Supplement: Supplementary Information — Supplementary Figures 1-4 and Supplementary Table 1 [file ncomms8104-s1.pdf]

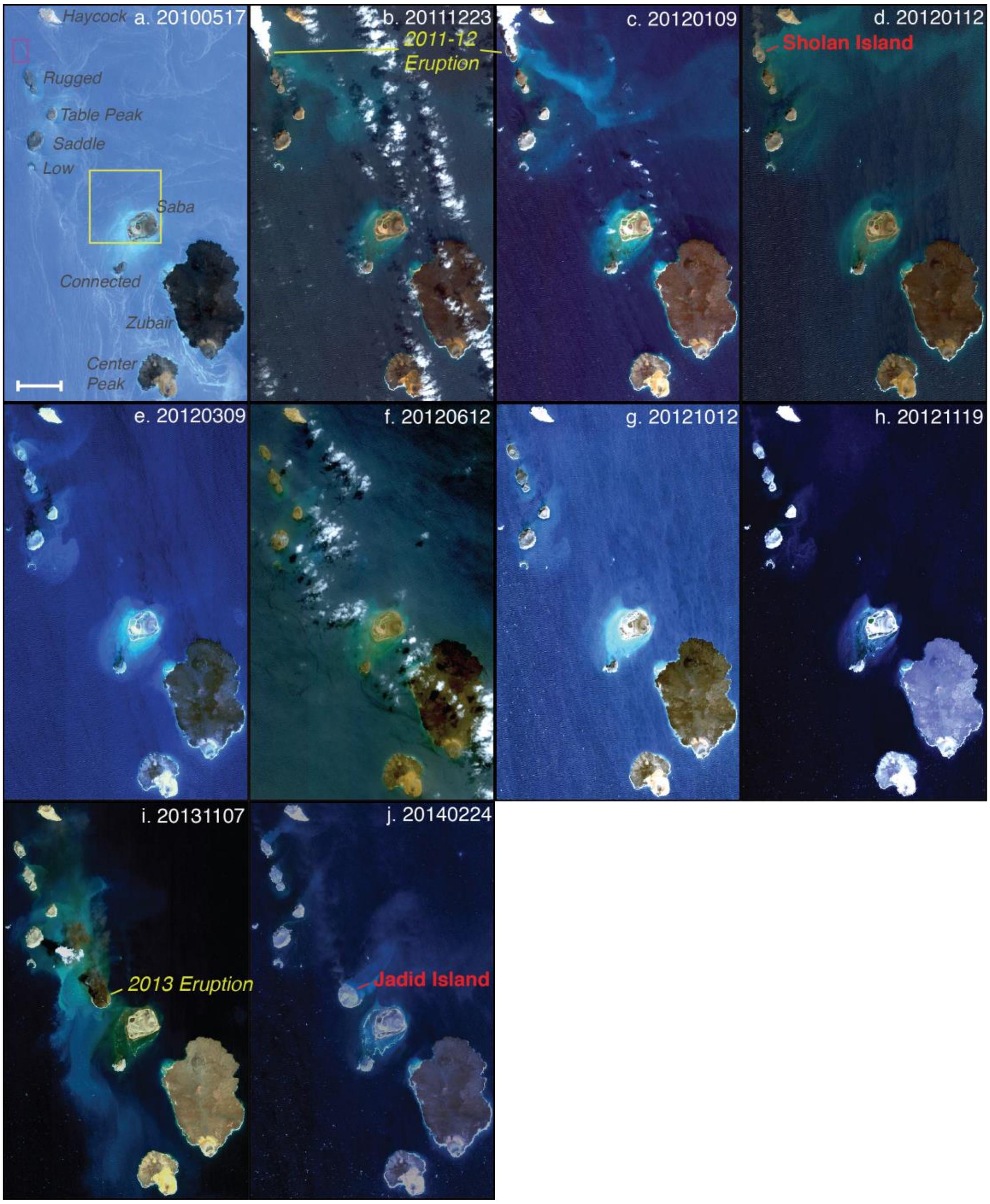

Supplementary Fig. 1. A series of natural color optical Quickbird & WorldView-2 preview images covering the Zubair archipelago. Images (a) and (b) show the 2011-12 Sholan eruption and image (i) shows the 2013 Jadid eruption. The purple and yellow rectangles in (a) mark the coverage of the optical images shown in Supplementary Fig. 2 and Supplementary Fig. 3, respectively. Scale bar in (a), 2 km.

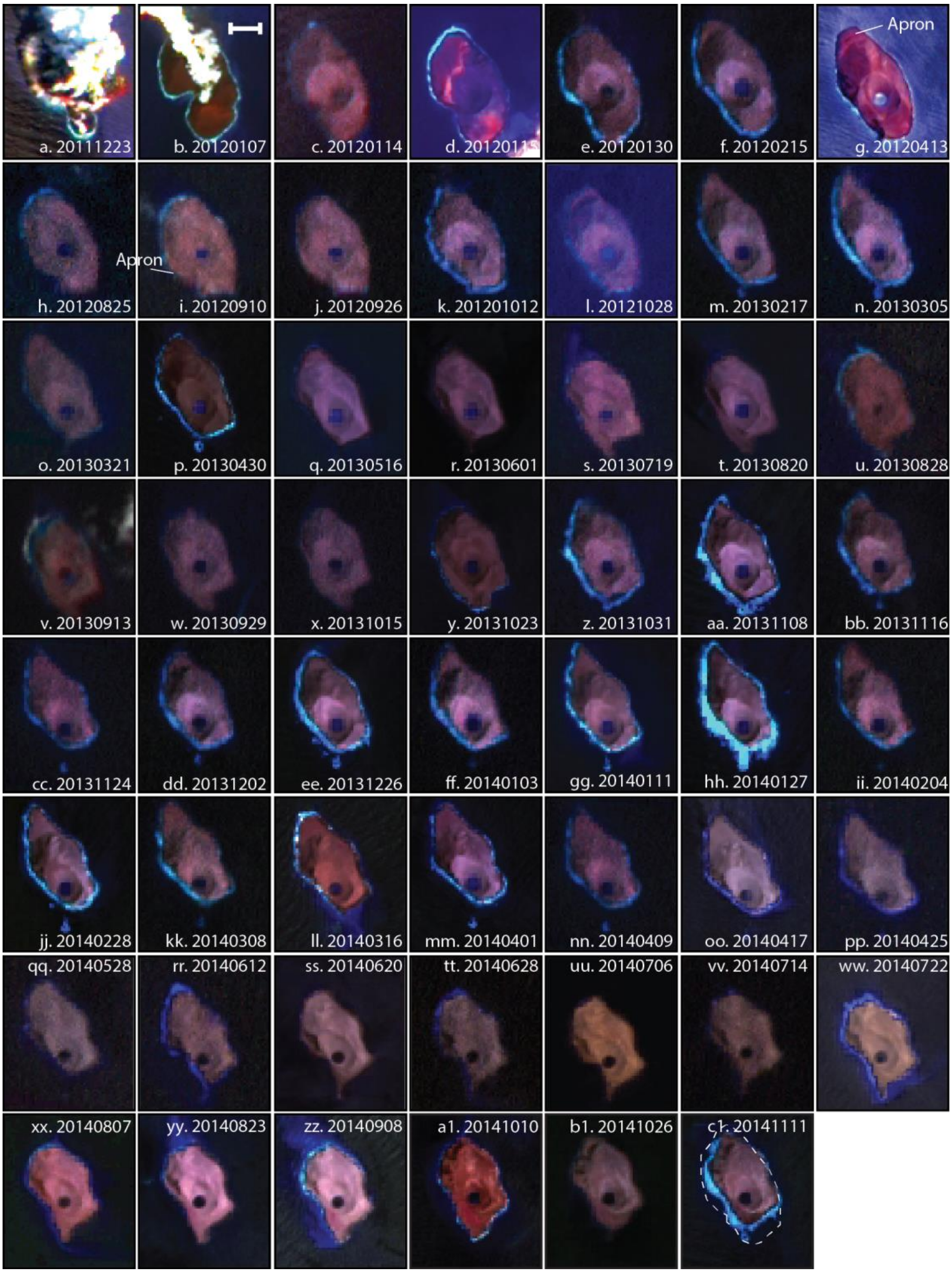

Supplementary Fig. 2. A series of false color satellite optical images showing the 2011-12 Sholan eruption and coastal changes of the island after the eruption ended. In general, the wind in the southern Red Sea varies seasonally with southerly winds between October and April and northerly winds from May to September. As a result a northern apron forms in winter and spring, while a western apron grows back in summer. The images are from EO-1 ALI (bands 10, 6, 4 and 1), Landsat-7 (bands 7, 4, 2 and 8), and Landsat-8 (bands 7, 5, 3 and 8). The white dashed line in (c1) shows the coastline from (c). Scale bar in (b), 200 m.

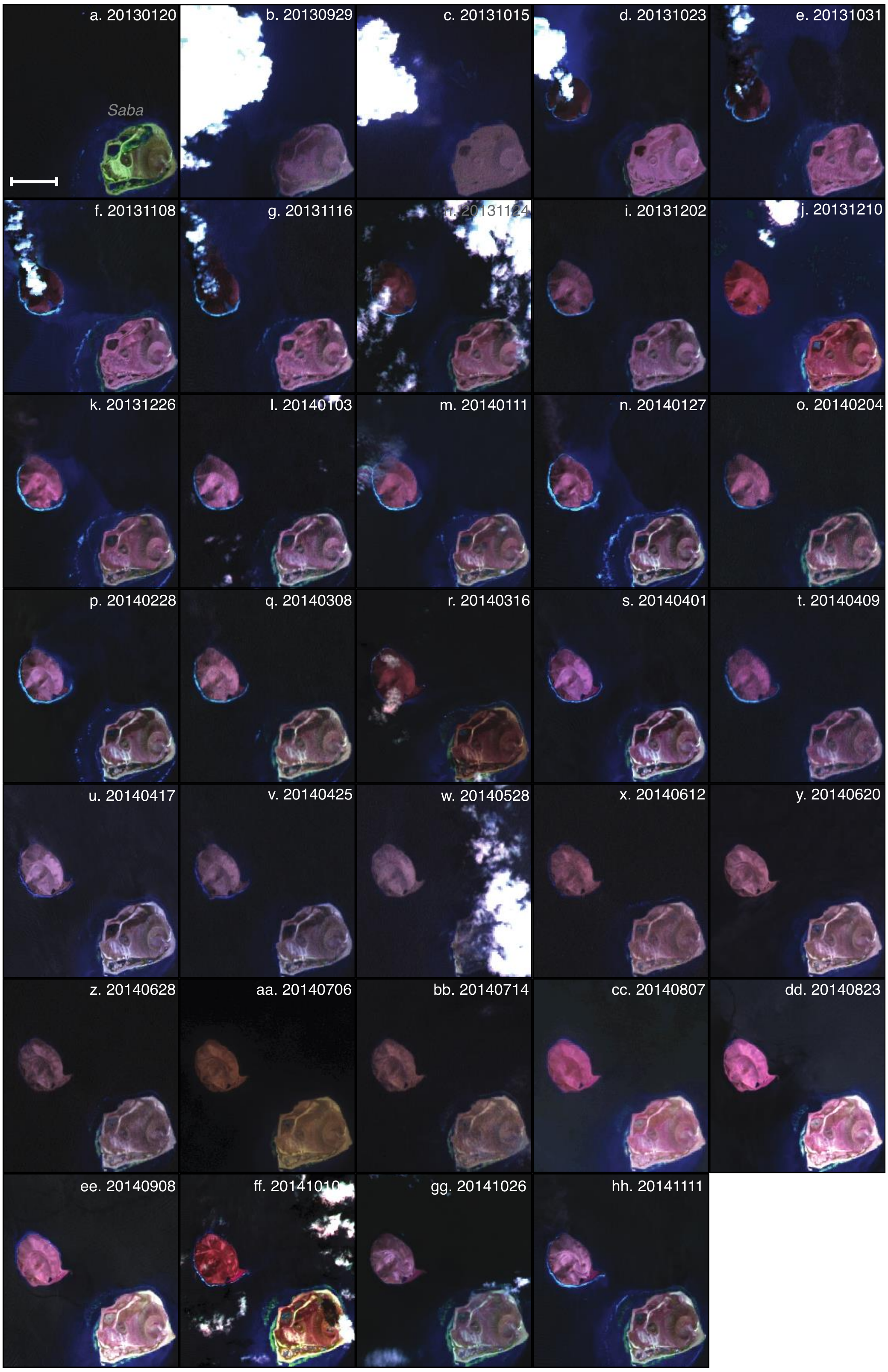

Supplementary Fig. 3. A series of false color optical images from the EO-1 ALI, Landsat-7 & 8 satellites of Jadid and Saba islands in the central Zubair archipelago, showing the 2013 eruption and post-eruptive coastal changes of Jadid Island. Scale bar in (a), 1 km.

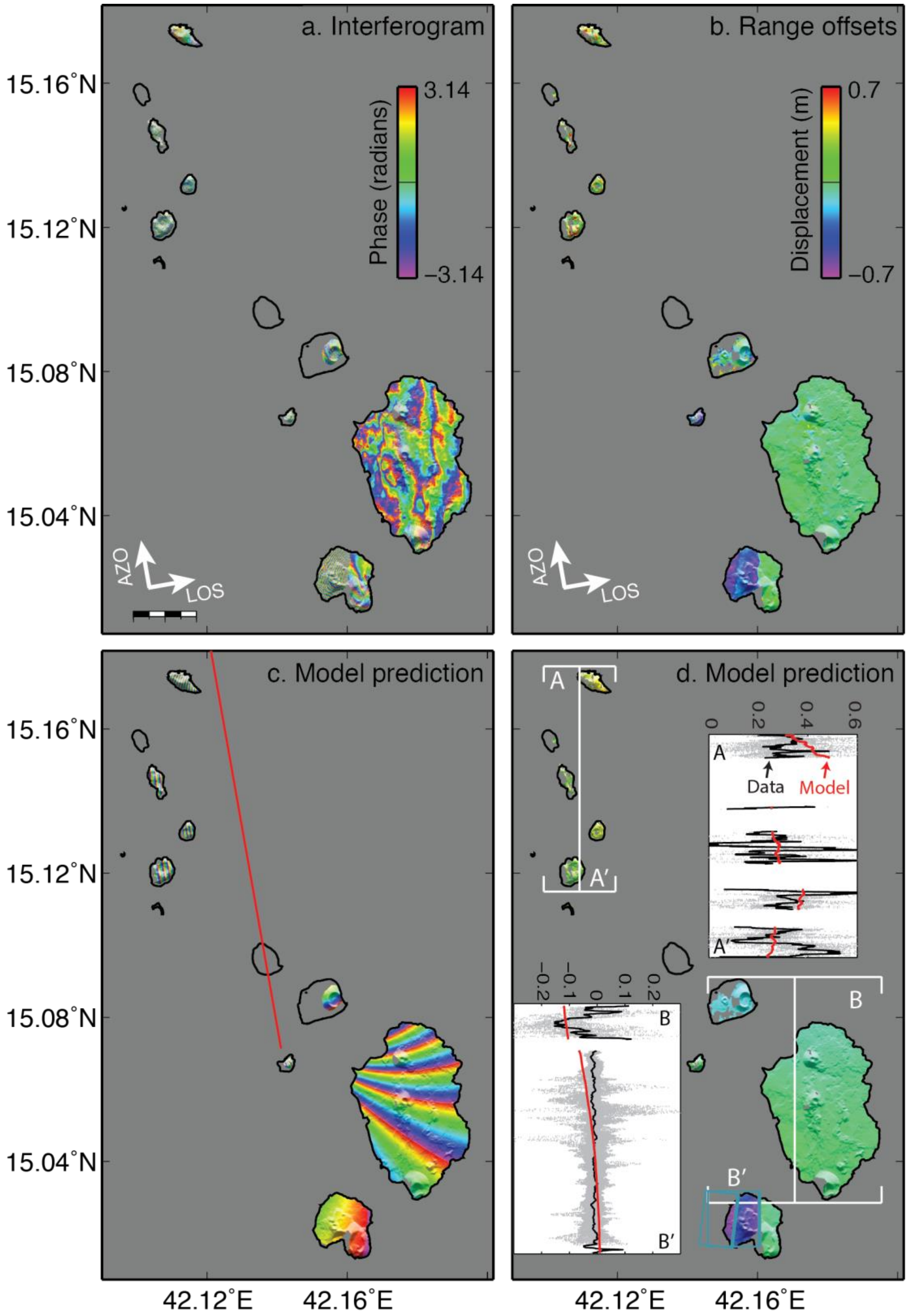

Supplementary Fig. 4. InSAR and offset images (from ascending orbit) of the Zubair archipelago showing deformation associated with 2013 Jadid eruption. (a), InSAR data. (b), Range offsets. (c-d), Model prediction for (a-b). The red line in (c) indicates the surface projection of the modeled dike under Jadid island. Insets in (d) show observed (black) and modeled (red) displacements along profiles A-A’ and B-B’. Positive range-offset values in (b) and (d) indicate ground movements up towards the satellite. Scale bar in (a), 2 km.

Satellite	Flight direction	Imaging mode	Orbit	Acq. Date (yyyymmdd)
TanDEM-X	Ascending	Stripmap	130	20111013<br>20121215<br>20131224
	Descending	Stripmap	46	20130820<br>20130922<br>20140212<br>20140328

Supplementary Table 1. Information about the TanDEM-X datasets.
